# Reasons for consultations and afflicted body systems in rural areas of The Republic of the Congo: A cross-sectional study

**DOI:** 10.1371/journal.pone.0333181

**Published:** 2025-10-17

**Authors:** Joseph Axel Ngatse, Gilbert Ndziessi, Ange C. Niama, Tristan M. Lepage, Jérémy T. Campillo, Sébastien D. Pion, François Missamou, Ange A. Abena, Michel Boussinesq, Cédric B. Chesnais

**Affiliations:** 1 Health Sciences Faculty, Marien NGOUABI University, Brazzaville, Republic of The Congo; 2 UMI233, French National Research Institute for Sustainable Development (IRD)-INSERM U1175- Montpellier University, Montpellier, France; 3 National Onchocerciasis Control Program, Brazzaville, Republic of The Congo; 4 Denis Sassou Nguesso University, Brazzaville, Republic of The Congo; Freelance Medical Research and Writing, UNITED KINGDOM OF GREAT BRITAIN AND NORTHERN IRELAND

## Abstract

In the Republic of the Congo, rural areas are characterized by vulnerable populations and endemic infectious diseases, while health facilities have limited technical capabilities. Our objective was to study the distribution of reasons for consultation and afflicted body systems in rural health facilities. We conducted a cross-sectional study in Bouenza, Lékoumou, and Sangha departments. Individuals attending General Medicine outpatient services of selected health facilities were included in the study between September 2020 and January 2021. Reasons for consultation and afflicted body systems were standardized using the second edition of the International Classification of Primary Care (ICPC-2). The overall results were subsequently stratified by department, sex, and age. Most participants were females (53.2%) and the most attended health facilities for consultation were health care centers (55.9%). The most common reason for consultation was fever (25.7%), followed by headache (8.9%), with the most common combination of reasons for consultation being ‘fever-cough-nasal congestion’ (4.5%). In Bouenza specifically, asthenia (3.0%) and headache (11.6%) were the most common reasons for consultation, whereas skin rash (4.6%) and foot injuries (2.0%) were the most frequent in Lekoumou and cough (8.1%) and chills (6.6%) the most frequent reasons for consultation in Sangha. Although neglected tropical diseases (NTDs) are considered to be common diseases in rural areas of the Republic of the Congo, reasons for consultation related to NTDs were uncommon in this study. According to the ICPC-2 classification, “fever or chills” (taken as non-specific manifestations, thus separate from body systems) and the digestive system were the most afflicted body systems, as observed in 29.7% and 21.2% of cases, respectively. The main reason for consultation was fever, and “fever or chills” was found to be the most frequent afflicted body system, followed by the digestive system. Further studies are required to complete the history of diseases, the medical diagnoses, and collect information during rainy seasons due to the seasonal nature of several diseases.

## Introduction

The epidemiological profile of a population is linked to the morbidity and mortality data available within its geographic region. These data sources collate field surveys, statistical reports generated by nations, and estimates provided by private and international organizations [1,2]. In Sub-Saharan Africa, challenges such as the suboptimal functioning of national health information systems and a dearth of high-quality research data publication in some countries have resulted in deficiencies within national health reports when it comes to producing valuable insights into disease prevalence [3–6]. These shortcomings could hinder countries from developing health policies based on evidence. It is therefore essential to estimate the epidemiological profile of populations to provide more informed guidance on health interventions.

The reasons for consultation, representing patients’ reported complaints to healthcare personnel, influence the course and outcome of medical procedures and inform the diagnostic process. Frequent medical diagnoses help define public health awareness and community screening programs.

Public health facilities in rural areas of the Republic of the Congo are facing significant challenges, including a shortage of qualified personnel, difficulties in accessing the facilities due to geographical and financial constraints, and inadequate diagnostic and treatment infrastructure [7]. In these settings, patient-reported signs and symptoms are often used to diagnose patients, particularly for many neglected tropical diseases (NTD) [8]. It is therefore vital to obtain high-quality data on the reasons for consultation in rural health facilities to gain a deeper understanding of the disease landscape. However, there is a lack of studies conducted in Congo on the distribution of reasons for consultation in rural health facilities settings. To address this gap in knowledge, we conducted a comprehensive investigation to evaluate the distribution of reasons for consultation and afflicted body systems in people living in rural areas in the Republic of the Congo.

## Methods

Data analyzed and the STROBE checklist for observational cross-sectional studies [9] are accessible in S1 and S2 Files, respectively.

### Study design and period

We conducted a multicenter, cross-sectional analytical study. The data collection period spanned from 23 September to 17 October 2020 for Lekoumou, from 11 September to 12 October 2020 for Bouenza, and from 17 December 2020–18 January 2021 for Sangha.

### Study setting

The study was conducted in public health posts and health centers, private hospitals, and public reference hospitals (also known as district hospitals) in three departments of the Republic of the Congo: Bouenza, Lékoumou and Sangha.

In the Republic of the Congo, the health system operates under a pyramid model with the health district as the operational level [10,11]. The health district (inhabitants: 30,000–100,000 in rural areas and 100,000–300,000 in urban areas) is comprised of a district hospital, also called a referral hospital, and an array of health facilities, including health posts, integrated health centers (inhabitants: 2,500–5,000 in rural areas and 5,000–10,000 in urban areas), and private hospitals (such as medical-social centers, medical offices). At the health district level of the national health pyramid, health posts are managed by community health workers or nurses, who refer patients to health centers for primary healthcare. Health centers and private hospitals are managed by nurses or general practitioners, who refer patients to district hospitals for specialized care, including cardiology and gastroenterology care.

The Bouenza department (capital: Madingou) covers an area of 12 265 km² and had an estimated population of 434 925 in 2018. The region features the Niari Valley, which is conducive to agriculture, and a humid tropical climate with corresponding temperatures ranging between 25°C and 30°C. The predominant vegetation includes herbaceous savannas, and plateaus dominate its topography [12]. The Bouenza department has 76 facilities (18 health posts, 16 health centers, 4 referral hospitals, and 38 private hospitals) and is endemic for soil-transmitted helminths (STH), urogenital schistosomiasis, onchocerciasis, lymphatic filariasis, Buruli ulcer [13], and Human African Trypanosomiasis [14]. The department is also empirically characterized by a high frequency of sickle cell disease, diabetes mellitus and hypertension [15].

The Lékoumou department (capital: Sibiti) covers an area of 20 950 km² with an estimated population of 135 643 in 2018. The region features the Chaillu granite massif, which is shared with Gabon, and has a humid tropical climate. The average annual rainfall is 2 200 mm, with temperatures ranging from 19.9°C in July to 24.5°C in March. The department’s vegetation includes vast, dense forests and shrubby savannahs in the south and north [16]. The Lékoumou department has 30 facilities (4 health posts, 21 health centers, 2 referral hospitals, and 3 private hospitals) and its endemic NTDs include leprosy, yaws, STH, onchocerciasis and schistosomiasis [13]. The department is also empirically characterized by a high frequency of sickle cell disease [15].

The Sangha department (capital: Ouesso) covers an area of 55 800 km² and had an estimated population of 120 650 in 2018. The department’s topography is characterized by a flat landscape with elevations consistently above 400 meters. The equatorial climate brings high and constant rainfall (1 600 mm annually), an average temperature of 26°C, and is characterized by a dense primary forests across the majority of the department [17]. The Sangha department has 41 facilities (8 health posts, 9 health centers, 1 referral hospital, and 23 private hospitals) and its endemic NTDs include leprosy, [18], yaws [19] and trachoma [20]. The department is also empirically characterized by a high frequency of hypertension [15].

### Sampling strategy and participant selection

A convenience sampling was used to only retain all rural health facilities and all referral hospitals in the health districts of the study departments. An initial selection phase was conducted between September and December 2019 in each department to identify the health facilities eligible for the study. This selection considered their location, the type of health facility, local ecology, and the documented distribution of NTDs in the respective areas. The selection of health facilities was based on two criteria: (i) their location in a rural area, situated more than 15 km away from the referral hospital (for health posts and health centers), and (ii) a minimum daily consultation rate of three patients, as indicated in health facilities’ registries. A total of 44 health facilities were selected according to selection criteria: 7 referral hospitals (4 in Bouenza, 2 in Lékoumou, and 1 in Sangha), 26 health centers (12 in Bouenza, 8 in Lékoumou, and 6 in Sangha), 4 private hospitals (3 medical-social centers and 1 clinic), and 7 health posts in Sangha. In comparison with the other two departments, Sangha had an insufficient number of health centers, which resulted in the selection of health posts and private facilities. The names and geographic coordinates of selected health facilities are presented in S3 File. To enhance clarity regarding abbreviations, a comprehensive list of these abbreviations is presented in S4 File.

Finally, we included all consenting patients who attended General Medicine outpatient services in the three selected departments during the study period. The consent provided was written in French and the two local languages (Lingala and Kituba). Participants who were recruited directly from the community instead of the health facility were excluded. The overall demographic composition of the study sample, representing age groups with a range of five years, reveals an overrepresentation of children under the age of five and females, compared with national population pyramids for 2018 and those of the three study departments, as produced by the National Statistics Institute (S5 File).

### Data collection

The data were collected prospectively for each patient during his/her consultation at each health facility by locally trained healthcare staff (community health workers or nurses for health posts, nurses or general practitioners for health centers, private hospitals, and general practitioners for reference hospitals). The data collection process at each respective health facility started for each health facility on the day the data collection tools were provided to the local health staff.

The initial version of the questionnaire was developed in 2019 and subsequently evaluated at 18 health facilities across the three departments towards the end of that same year. Following the receipt of feedback from health personnel, a revised version was developed at the outset of 2020. However, due to the onset of the COVID-19 pandemic, travel to the departments was suspended during this period. Consequently, this new updated version was exclusively assessed at health facilities located on the outskirts of Brazzaville. The definitive version of the questionnaire was officially validated in August 2020. The questionnaire gathered general consultation variables derived from the curative consultation records at the health centers visited. The initial section of the questionnaire documented the reasons for consultation, as reported by the patients (see S6 File).

Data was collected using paper-based questionnaires. This choice was driven by logistical constraints: purchasing electronic tablets for each of the 44 selected health facilities was not cost-effective, and in rural areas, the lack of electricity would have limited the use of electronic devices due to battery depletion. Data collection was prospectively completed by trained local healthcare professionals during patient consultations. We collected data on participants’ demographics (age, sex, occupation), department, type of health facility for consultation, reasons for consultation, and afflicted body systems. Standard body system categories were used (e.g., “abdominal”), with the addition of two separate systems: “general”, for reasons that could not be categorized within a specific body system, and “fever or chills”, designed to reduce the frequency of these two reasons being reported under the “general” system.

Given the variability in the number of reasons for consultation reported by patients, each of the six reasons provided was treated as a separate variable. To ensure consistency in documenting reasons for consultation and affected body systems, we used the International Classification of Primary Care, second edition [21,22]. This classification system, developed in 1998 by the “World Organization of National Colleges, Academies, and Academic Associations of General Practitioners/Family Physicians”, enables comparability across study sites – most of which were primary healthcare facilities (health posts and health centers) – and with other studies conducted in African settings. In ICPC-2, each reason for consultation is coded with a letter representing the afflicted body system, followed by two numbers indicating the specific complaint/reason for consultation [23].

The data from the paper questionnaires were entered into an electronic format using the REDCap mobile application (www.projectredcap.org), a tool developed by Vanderbilt University (Nashville, Tennessee, USA) [24,25]. To ensure accuracy and minimize errors, trained interviewers conducted a double-entry process. Furthermore, the geographic coordinates (longitude and latitude) for each study site were collected using the Global Positioning System smartphone application.

### Statistical analysis

The data collected on REDCap was exported to Microsoft Excel 2016 (version 16.0) from Microsoft Office, where it was cleaned. Subsequently, the data was exported for statistical analyses on R 4.0.3 software, utilizing the RStudio 2022.02.03 interface.

As a first step in variable construction, we examined the sequence in which patients reported their reasons for consultation. Accordingly, “reason 1” (R_1_) was defined as the first reason for consultation mentioned by the patient, while “system 1” (S_1_) represented the afflicted body system corresponding to R_1_. The variables R_1-3_ and S_1-3_ referred to the combination of the first three reasons for consultation and their afflicted body systems that a patient expressed, respectively. In a second step, we created the variable “all reasons for consultation”, encompassing all reasons for consultation mentioned by a given patient, regardless of order. Similarly, “all body systems” included all afflicted body systems related to “all reasons for consultation”, without regard to order.

For descriptive statistical analysis, categorical variables were summarized as frequencies and percentages. To compare proportions between groups, we used the Chi-square test. Fisher’s exact test was applied when expected cell counts were below 5 or when assumptions for the Chi-square test were not met. Age was treated as a categorical variable. A two-sided p-value of <0.05 was considered to denote statistically significant results. For each proportion, 95% confidence intervals (95% CI) were computed using either the Wald when np ≥ 5 and n(1 − p)≥5 or Wilson method [26,27]. No statistical test was performed to compare the frequencies of the different combinations of reasons for consultation (R1–R3) as well as first combinations of afflicted body systems, as each combination represented a unique, non-mutually exclusive group. These frequencies are presented descriptively, along with their 95% confidence intervals, to illustrate the most frequently reported patterns.

The results were presented for the overall study population, then stratified by sex, department, and age group. All the most common first reasons for consultation and afflicted body systems were presented, as reported by patients from each study department (see S7 File for description).

### Ethical issues

The ethical clearance was obtained from the Health Sciences Research Ethics Committee (CERSSA, in French) on 29 July 2019, under the reference N°166/MRSIT/IRSSA/CERSSA, for a broader study entitled “Assessment of the Burden of Neglected Tropical Diseases in Rural Areas of the Republic of the Congo”. This article is the first in a series of publications on neglected tropical diseases, and a unique sampling and data collection strategy was employed. The study was conducted in accordance with the principles of the Helsinki Declaration [28].

## Results

### Sociodemographic characteristics of the study population

The study revealed that the largest age group of participants was those aged 15–29 (24.1%), while females (53.2%) and students (20.5%) were the most frequent (Table 1).

**Table 1:** Study sites and characteristics of the 1876 patients.

Variable	Categories	n	%
**Department**	Bouenza	712	37.9
	Lékoumou	350	18.7
	Sangha	814	43.4
**Type of health facilities**	Health post	218	11.6
	Health center	1 048	55.9
	Private hospital	195	10.4
	Reference hospital	415	22.1
**Age**	0-4	404	21.5
	5-14	275	14.7
	15-29	452	24.1
	30-49	439	23.4
	50+	306	16.3
**Sex**	Female	998	53.2
	Male	878	46.8
**Occupation**	Farmer	333	17.7
	Housewife	251	13.4
	Student	385	20.5
	Out-of-school infants	324	17.3
	No occupation	243	13.0
	Driver	26	1.4
	Fisher	21	1.1
	Retired	15	0.8
	Dressmaker	14	0.7
	Mechanic	10	0.5
	Other*	254	13.5

* The term ‘other’ is used to denote occupations for which there were fewer responses.

### Reasons for consultation

#### Overall distribution.

According to the ICPC-2 classification, the distribution of R_1_ indicates that 25.6% (95% CI: 23.7–27.6) of the subjects reported having a fever, with the percentage being consistent across all departments (Table 2). The most frequent R_1-3_ combination (Table 3) was “fever-cough-nasal congestion” (4.5%, 95% CI: 3.5–5.8), followed by “fever-asthenia-anorexia” (2.2%, 95% CI: 1.5–3.3).

**Table 2:** Top 28 first reasons for consultation. N = 1867.

	ICPC-2 code	Bouenza (n = 712)		Lékoumou (n = 350)		Sangha (n = 814)			Overall (n = 1 876)	
First reasons for consultation (R_1_)		n	% and 95%CI	n	% and 95%CI	n	% and 95%CI	p	n	% and 95%CI
Fever	A03	166	23.3 (20.2–26.4)	90	25.7 (21.1–30.3)	224	27.5 (24.4–30.6)	0.171	480	25.6 (23.7–27.6)
Headache	N01	82	11.5 (9.2–13.8)	25	7.1 (4.4–9.8)	59	7.2 (5.4–9.0)	0.006	166	8.8 (7.6–10.2)
Generalized abdominal pain	D01	35	4.9 (3.3–6.5)	27	7.7 (4.9–10.5)	58	7.1 (5.3–8.9)	0.114	120	6.4 (5.3–7.6)
Cough	R05	27	3.8 (2.4–5.2)	12	3.4 (1.5–5.3)	66	8.1 (6.2–10.0)	<0.005	105	5.6 (4.7–6.7)
Diarrhea	D11	38	5.3 (3.7–6.9)	5	1.4 (0.2–2.6)	41	5.0 (3.5–6.5)	0.009	84	4.5 (3.7–5.5)
Chills	A02	13	1.8 (0.8–2.8)	7	2.0 (0.5–3.5)	54	6.6 (4.9–8.3)	<0.005	74	3.9 (3.1–4.8)
Other localized abdominal pain	D06	25	3.5 (2.2–4.8)	14	4.0 (1.9–6.1)	25	3.1 (1.9–4.3)	0.713	64	3.4 (2.6–4.3)
Vomiting	D10	21	2.9 (1.7–4.1)	3	0.9 (0.1–1.9)	25	3.1 (1.9–4.3)	0.073	49	2.6 (1.9–3.4)
Localized rash	S06	14	2.0 (1.0–3.0)	16	4.6 (2.4–6.8)	16	2.0 (1.0–3.0)	0.018	46	2.5 (1.8–3.3)
Other traumatic skin injury	S19	16	2.2 (1.1–3.3)	12	3.4 (1.5–5.3)	18	2.2 (1.2–3.2)	0.424	46	2.5 (1.8–3.3)
Dizziness	N17	22	3.1 (1.8–4.4)	5	1.4 (0.2–2.6)	18	2.2 (1.2–3.2)	0.033	45	2.4 (1.7–3.2)
Generalized weakness	A04	21	2.9 (1.7–4.1)	4	1.1 (0.0–2.2)	10	1.2 (0.5–1.9)	0.059	35	1.9 (1.3–2.6)
Lumbar symptoms/complaints	L03	12	1.7 (0.8–2.6)	5	1.4 (0.2–2.6)	13	1.6 (0.7–2.5)	0.759	30	1.6 (1.1–2.3)
Pruritus	S02	14	2.0 (1.0–3.0)	3	0.9 (0.1–1.9)	10	1.2 (0.5–1.9)	0.429	27	1.4 (0.9–2.1)
Dyspnea	R02	11	1.5 (0.6–2.4)	3	0.9 (0.1–1.9)	11	1.4 (0.6–2.2)	0.729	25	1.3 (0.8–2.0)
Epigastric pain	D02	10	1.4 (0.5–2.3)	5	1.4 (0.2–2.6)	9	1.1 (0.4–1.8)	0.561	24	1.3 (0.8–1.9)
Joint symptoms/complaints	L20	14	2.0 (1.0–3.0)	2	0.6 (0.2–1.4)	7	0.9 (0.3–1.5)	0.159	23	1.2 (0.7–1.9)
Foot symptoms/complaints	L17	5	0.7 (0.1–1.3)	7	2.0 (0.5–3.5)	10	1.2 (0.5–1.9)	0.199	22	1.2 (0.7–1.8)
Chest pain	A11	10	1.4 (0.5–2.3)	6	1.7 (0.4–3.0)	4	0.5 (0.0–1.0)	0.234	20	1.1 (0.7–1.7)
Convulsion	N07	10	1.4 (0.5–2.3)	2	0.6 (0.2–1.4)	7	0.9 (0.3–1.5)	0.258	19	1.0 (0.6–1.6)
Nasal congestion	R07	15	2.1 (1.0–3.2)	1	0.3 (0.3–0.9)	2	0.2 (0.1-0.5)	<0.005	18	1.0 (0.6–1.5)
Skin swelling	S04	7	1.0 (0.3–1.7)	6	1.7 (0.4–3.0)	5	0.6 (0.1–1.1)	0.276	18	1.0 (0.6–1.5)
Dysuria	U01	5	0.7 (0.1–1.3)	6	1.0 (0.0–2.0)	7	0.9 (0.3–1.5)	0.448	18	1.0 (0.6–1.5)
Loss of appetite	T03	5	0.7 (0.1–1.3)	1	0.3 (0.3–0.9)	8	1.0 (0.3–1.7)	0.434	14	0.7 (0.4–1.2)
Leg symptoms/complaints	L14	8	1.1 (0.3–1.9)	2	0.6 (0.2–1.4)	3	0.4 (0.0–0.8)	0.209	13	0.7 (0.4–1.2)
Muscle pain	L18	3	0.4 (0.1–0.9)	4	1.1 (0.0–2.2)	6	0.7 (0.1–1.3)	0.720	13	0.7 (0.4–1.2)
Intermenstrual bleeding	X08	7	1.0 (0.3–1.7)	3	0.9 (0.1–1.9)	3	0.4 (0.0–0.8)	0.474	13	0.7 (0.4–1.2)
Hematuria	U06	9	1.3 (0.5–2.1)	1	0.3 (0.3–0.9)	1	0.1 (0.1–0.3)	0.057	11	0.6 (0.3–1.1)
Other		83	11.7 (9.3–14.1)	68	19.4 (15.4–23.4)	94	11.5 (9.3–13.7)	0.012	245	13.1(11.7–14.6)

ICPC: International Classification of Primary Care. CI: confidence interval.

**Table 3:** Most frequent combinations of the first three reasons for consultation (N = 1110).

Reason combinations (R_1_-R_2_-R_3_)	n	% and 95% CI
Fever – Cough – Nasal congestion	50	4.5 (3.5–5.8)
Fever – Generalized weakness – Loss of appetite	24	2.2 (1.5–3.3)
Fever – Generalized abdominal pain – Vomiting	20	1.8 (1.2–2.8)
Fever – Generalized abdominal pain – Cough	19	1.7 (1.1–2.7)
Fever – Vomiting – Headache	18	1.6 (1.0–2.6)
Chills – Fever – Headache	17	1.5 (0.9–2.5)
Fever – Generalized weakness – Vomiting	17	1.5 (0.9–2.5)
Fever – Vomiting – Diarrhea	17	1.5 (0.9–2.5)
Generalized weakness – Vomiting – Diarrhea	17	1.5 (0.9–2.5)
Fever – Generalized weakness – Headache	16	1.4 (0.9–2.4)
Fever – Dyspnea – Cough	16	1.4 (0.9–2.4)
Fever – Headache – Cough	15	1.4 (0.8–2.3)
Fever – Muscle pain – Headache	14	1.3 (0.8–2.2)
Fever – Headache – Dizziness	14	1.3 (0.8–2.2)
Fever – Generalized abdominal pain – Headache	13	1.2 (0.7–2.1)
Fever – Joint – Headache	13	1.2 (0.7–2.1)
Fever – Generalized abdominal pain – Diarrhea	11	1.0 (0.6–1.9)
Fever – Vomiting – Cough	11	1.0 (0.6–1.9)
Fever – Headache – Loss of appetite	9	0.8 (0.4–1.6)
Chills – Fever – Generalized weakness	8	0.7 (0.4–1.5)
Chills – Generalized abdominal pain – Headache	8	0.7 (0.4–1.5)
Fever – Diarrhea – Cough	8	0.7 (0.4–1.5)
Asthenia – Headache – Dizziness	8	0.7 (0.4–1.5)
Chills – Fever – Loss of appetite	7	0.6 (0.3–1.4)
Chills – Joint – Headache	7	0.6 (0.3–1.4)
Chills – Headache – Dizziness	7	0.6 (0.3–1.4)
Fever – Generalized weakness – Cough	7	0.6 (0.3–1.4)
Fever – Headache – Nasal congestion	7	0.6 (0.3–1.4)
Chills – Headache – Loss of appetite	6	0.5 (0.2–1.3)
Generalized weakness – Diarrhea – Loss of appetite	6	0.5 (0.2–1.3)
Generalized abdominal pain – Vomiting – Diarrhea	6	0.5 (0.2–1.3)
Vomiting – Diarrhea – Cough	6	0.5 (0.2–1.3)
Headache – Dizziness – Loss of appetite	6	0.5 (0.2–1.3)
Fever – Generalized weakness – Generalized abdominal pain	5	0.5 (0.2–1.2)
Fever – Generalized weakness – Diarrhea	5	0.5 (0.2–1.2)
Fever – Generalized weakness – Dizziness	5	0.5 (0.2–1.2)
Fever – Chest pain – Cough	5	0.5 (0.2–1.2)
Fever – Vomiting – Loss of appetite	5	0.5 (0.2–1.2)
Fever – Lumbar symptoms and complaints – Headache	5	0.5 (0.2–1.2)
Fever – Cough – Loss of appetite	5	0.5 (0.2–1.2)
Headache – Cough – Nasal congestion	5	0.5 (0.2–1.2)
Other	642	57.8 (54.9–60.6)

Each combination represents the first three reasons for consultation recorded per patient. Percentages and 95% confidence intervals are shown. No statistical comparison was performed between combinations.

The sample size for combinations of reasons for consultation is reduced (from n = 1867 to n = 1110) because we only retained patients who expressed at least three reasons for consultation.

#### Distribution by sex and department.

Regarding R_1_, patients in Bouenza reported a higher prevalence of headaches (11.6%) and asthenia (3.0%) compared with those in Lékoumou (7.2% and 1.2% respectively, p = 0.006) and Sangha departments (7.2% and 1.2% respectively, p = 0.025). In contrast, patients in Lékoumou had rashes more frequently (4.6%), compared with those in Bouenza and Sangha (2.0% and 2.0% respectively, p = 0.016). Conversely, the proportion of diarrhea was notably lower in Lékoumou (1.4%) compared with Bouenza and Sangha (5.4% and 5.0%, respectively) (p = 0.010). Additionally, patients in Sangha reported a higher frequency of cough (8.1%) and chills (6.6%) compared with those in Bouenza (3.8% and 1.8% respectively, p < 0.001) and Lékoumou (3.5% and 2.0% respectively, p < 0.001).

**Fig 1:**
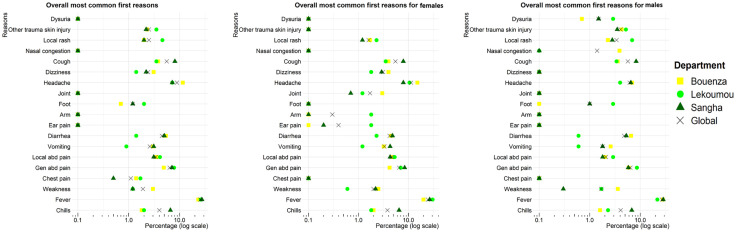
Distribution of the first reasons for consultation stratified by sex and department.

The findings on R_1_ regarding headache, rash, cough, and chills were consistent within each sex and displayed similar patterns between both sexes. Specifically, joint complaints were more frequent in Bouenza (3.0%) than in Lékoumou (1.2%) or Sangha (0.7%) (p = 0.032). Conversely, complaints about arm issues were exclusively reported in Lékoumou (1.8%, p = 0.006), while ear pain was more frequent in Lékoumou (1.8%) than in Bouenza (0.0%) or Sangha (0.2%) (p = 0.020) (Fig 1).

#### Distribution by sex and age groups.

**Fig 2:**
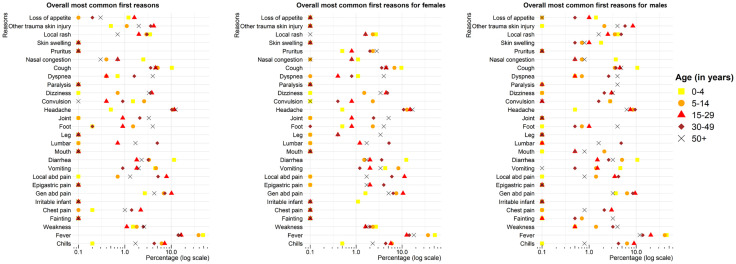
Distribution of the first reasons for consultation stratified by sex and age group.

The distribution of R_1_ by age group (Fig 2) revealed that children aged 0–4 years had the highest proportions of cough (10.3%) and diarrhea (11.5%), in comparison with children aged 5–14 years (5.1% and 3.3%, respectively), 15–29-year-old individuals (4.6% and 1.8%, respectively), 30–49-year-old individuals (3.6% and 3.2%, respectively), and those aged 50 years and over (4.6% and 2.3% respectively, p < 0.001). The highest proportions of seizures were observed in individuals aged 5–14 years (2.6%), while the percentages were lower in the 0–5 years, 15–29 years, 30–49 years, and 50 years and over (1.5%, 0.4%, 0.9%, and 0.0% respectively, p = 0.014). In the case of generalized or localized abdominal pain, individuals aged 15–29 years had the highest proportions (10.1% and 7.9%, respectively), while lower percentages were observed in the 0–4-year-old (2.7% and 0.0%, respectively), 5–14-year-old (6.9% and 0.7%, respectively), 30–49-year-old (7.3% and 5.2%, respectively), and 50 years and over (4.3% and 1.3% respectively, p < 0.001). For low back pain, the 30–49-year-olds had the highest proportions (5%), compared with the 0–4-year-olds, 5–14-year-old, 15–29-year-old, and 50 years and over (0.0%, 0.0%, 0.7%, and 1.7%, respectively, p < 0.001). Furthermore, individuals aged 50 years and over had the highest proportions of dyspnea (3.9%) and joint complaints (3.3%), compared with 0–4-year-old (0.7% and 0.0%, respectively), 5–14-year-old (0.4% and 0.0%, respectively), 15–29-year-old (0.4% and 0.9%, respectively) and 30–49-year-old age groups (1.6% and 2.1%, respectively) (p < 0.001 for both dyspnea and join complaints).

Additionally, several reasons for consultation were only reported by females: joint complaints were more prevalent in those aged 50 years and over (5.1%) when compared with the 0–4-year-old, 5–14-year-old, 15–29-year-old, and 30–49-year-old age groups (0.0%, 0.0%, 0.8%, and 2.4%, respectively, p = 0.001). Complaints about leg issues were more frequent in those aged 50 years and over (3.4%), compared with the 0–4-year, 5–14-year, 15–29 year, and 30–49-year age groups (0.0%, 0.0%, 0.4%, and 0.4% respectively, p = 0.004). Epigastric pain was more frequent in those aged 30–49 years (4.0%) compared with the 0–4-year, 5–14-year, 15–29 year, and 50 years and over age groups (0.0%, 0.0%, 2.0%, and 1.7% respectively, p = 0.008).

Several reasons for consultation were exclusively reported by males. These included the loss of appetite, which was more frequent in children aged 0–4 years (1.4%), compared with the 5–14 year, 15–29-year, 30–49 year, and 50 years and over age groups (0.0%, 1.0%, 0.5%, and 0.0% respectively, p = 0.012). Additionally, traumatic skin injuries were more frequent among those aged 15–29 years (8.5%), compared with those aged 0–4 years, 5–14 years, 30–49 years, and 50 years and over (0.0%, 2.1%, 5.8% and 4.0% respectively, p < 0.001). Paralysis was exclusively reported by patients aged 50 years and over (4.0%, p < 0.001), fainting (loss of consciousness) was more common in the 50 years and over (3.2%), compared with the 0–4-year, 5–14-year, 15–29 year and 30–49 year age groups (0.0%, 0.7%, 0.0%, and 0.5% respectively, p = 0.004).

The distribution for each department of the first reasons for consultation, stratified by sex and age (in S8 File), shows proportions higher than 10% of fever for all age groups in all departments. Additionally, the presence of distinct R_1_ with proportions exceeding 10% is observed in the 0–4-year-old age group, including diarrhea in Bouenza (15.4%), localized rash in Lékoumou (10.9%), and cough in Sangha (16.5%). For reasons for consultation considered regardless of their order of expression (in S9 File), the highest proportions were observed for headaches in Bouenza, for the 5–14, 15–29, 30–49 and 50 years and over (12.5%, 12.5%, 11.2%, 12.7%, respectively). Additionally, cough was identified as most frequent in Lékoumou and Sangha in children aged 0–4 years (12.6% and 18.0% respectively). Finally, the “fever-cough-nasal congestion” combination (in S10 File) was observed to be more frequent in Sangha (6.7%), and in 0–4-year-olds (16.2%).

### Afflicted body systems

#### Overall distribution.

The most frequently afflicted body systems related to first expressed reasons for consultation were “Fever or chills” (29.7%, 95% CI: 23.7–27.6), followed by digestive (21.2%, 95% CI: 19.3–23.0), neurological (13.0% 95% CI: 11.5–14.4), and respiratory (8.5%, 95% CI: 7.3–9.8) systems (Table 4). Making a correspondence between each of the first reasons for consultation and a body system, the most frequent combination of systems was “ Respiratory – Respiratory – Respiratory” (3.8%), followed by “Digestive – Neurological – Fever or chills” (3.4%) (Table 5).

**Table 4:** Afflicted body systems according to first reasons for consultation. N = 1867.

	ICPC-2 code	Bouenza (n = 712)		Lékoumou (n = 350)		Sangha (n = 814)			Overall (n = 1 876)	
First afflicted body systems		n	% and 95% CI	n	% and 95% CI	n	% and 95% CI	p	n	% and 95% CI
Fever or chills	ZZ	179	25.1 (22.0–28.3)	97	27.7 (23.0–32.4)	278	34.2 (30.9–37.4)	0.001	554	29.5 (27.5–31.5)
Digestive	D	136	19.1 (16.2–22.0)	73	20.9 (16.6–25.1)	186	22.9 (20.0–25.7)	0.054	395	21.1 (19.3–23.0)
Neurological	N	119	16.7 (14.0–19.5)	38	10.9 (7.6–14.1)	85	10.4 (8.3–12.5)	<0.005	242	12.9 (11.5–14.4)
Respiratory	R	60	8.4 (6.4–10.5)	18	5.1 (2.8–7.5)	81	10.0 (7.9–12.0)	<0.005	159	8.5 (7.3–9.8)
Skin	S	59	8.3 (6.3–10.3)	40	11.4 (8.1–14.8)	58	7.1 (5.4–8.9)	0.018	157	8.4 (7.2–9.7)
Osteoarticular	L	48	6.7 (4.9–8.6)	30	8.6 (5.6–11.5)	47	5.8 (4.2–7.4)	0.420	125	6.7 (5.7–7.9)
General	A	40	5.6 (3.9–7.3)	11	3.1 (1.3–5.0)	25	3.1 (1.9–4.3)	0.110	76	4.1 (3.3–5.1)
Urinary	U	20	2.8 (1.6–4.0)	9	2.6 (0.9–4.2)	11	1.4 (0.6–2.1)	0.150	40	2.1 (1.6–2.9)
Female genital	X	13	1.8 (0.8–2.8)	7	2.0 (0.5–3.5)	9	1.1 (0.4–1.8)	0.670	29	1.5 (1.0–2.2)
Metabolic	T	10	1.4 (0.5–2.3)	5	1.4 (0.2–2.7)	9	1.1 (0.4–1.8)	0.890	24	1.3 (0.9–2.0)
Eye	F	9	1.3 (0.4–2.1)	3	0.9 (0.1–1.8)	8	1.0 (0.3–1.7)	0.720	20	1.1 (0.7–1.7)
Ear	H	3	0.4 (0.1–0.9)	7	2.0 (0.5–3.5)	6	0.7 (0.1–1.3)	0.040	16	0.9 (0.6–1.5)
Cardiovascular	K	6	0.8 (0.2–1.5)	3	0.9 (0.1–1.8)	6	0.7 (0.1–1.3)	0.880	15	0.8 (0.5–1.4)
Male genital	Y	2	0.3 (0.1–0.7)	3	0.9 (0.1–1.8)	2	0.2 (0.1–0.6)	0.490	7	0.4 (0.2–0.9)
Psychological	P	1	0.1 (0.1–0.4)	0	0.0 (0.0–0.0)	3	0.4 (0.0–0.8)	0.290	4	0.2 (0.1–0.7)
Blood	B	2	0.3 (0.1–0.7)	1	0.3 (0.3–0.8)	0	0.0 (0.0–0.0)	0.250	3	0.2 (0.1–0.6)
Family planning	W	1	0.1 (0.1–0.4)	0	0.0 (0.0–0.0)	0	0.0 (0.0–0.0)	0.610	1	0.1 (0.0–0.5)

For each proportion, 95% confidence intervals (95% CI) were computed using either the Wald or Wilson method.

**Table 5:** Most frequent triplets of afflicted body systems according to the triplets of first reasons for consultation in each patient. N = 1110.

System combinations (S_1_-S_2_-S_3_)	n	% and 95% CI
Respiratory – Respiratory – Respiratory	71	6.4 (5.0-7.8)
Digestive – Neurological – Fever or chills	69	6.2 (4.8-7.6)
Digestive – Digestive – Fever or chills	64	5.8 (4.4-7.1)
Digestive-Respiratory- Fever or chills	57	5.1 (3.8-6.4)
Osteoarticular-Neurological- Fever or chills	49	4.4 (3.2-5.6)
General-Digestive- Fever or chills	38	3.4 (2.4-4.5)
General -Metabolic- Fever or chills	31	2.8 (1.8-3.8)
General -Neurological- Fever or chills	30	2.7 (1.7-3.7)
Digestive-Digestive-Digestive	30	2.7 (1.7-3.7)
Neurological-Respiratory- Fever or chills	27	2.4 (1.7-3.5)
General -Digestive-Digestive	25	2.3 (1.5-3.3)
Digestive- Metabolic – Fever or chills	24	2.2 (1.5-3.2)
Neurological-Neurological- Fever or chills	23	2.1 (1.4-3.1)
Neurological- Fever or chills – Fever or chills	19	1.7 (1.1-2.7)
General -Respiratory- Fever or chills	17	1.5 (1.0-2.4)
Neurological – Metabolic – Fever or chills	16	1.4 (0.9-2.3)
Digestive – Digestive – Neurological	15	1.4 (0.8-2.2)
Digestive – Digestive – Respiratory	15	1.4 (0.8-2.2)
Digestive – Osteoarticular – Neurological	15	1.4 (0.8-2.2)
General – Digestive – Metabolic	14	1.3 (0.8-2.1)
Digestive – Osteoarticular – Fever or chills	13	1.2 (0.7-2.0)
Digestive – Respiratory – Respiratory	12	1.1 (0.6-1.9)
Osteoarticular – Respiratory – Fever or chills	12	1.1 (0.6-1.9)
Osteoarticular – Skin – Skin	12	1.1 (0.6-1.9)
General – Digestive – Neurological	11	1.0 (0.6-1.8)
General – Neurological – Neurological	11	1.0 (0.6-1.8)
Digestive – Fever or chills – Fever or chills	11	1.0 (0.6-1.8)
Respiratory – Metabolic – Fever or chills	10	0.9 (0.5-1.7)
General – Neurological – Respiratory	9	0.8 (0.4-1.5)
Digestive – Digestive – Metabolic	9	0.8 (0.4-1.5)
Osteoarticular – Neurological – Neurological	9	0.8 (0.4-1.5)
General – Fever or chills – Fever or chills	8	0.7 (0.4-1.4)
Osteoarticular – Osteoarticular – Fever or chills	8	0.7 (0.4-1.4)
Neurological – Respiratory – Respiratory	8	0.7 (0.4-1.4)
Metabolic – Fever or chills – Fever or chills	8	0.7 (0.4-1.4)
Osteoarticular – Neurological – Respiratory	7	0.6 (0.3-1.3)
Respiratory – Skin – Fever or chills	7	0.6 (0.3-1.3)
General – Digestive – Respiratory	6	0.5 (0.2-1.2)
Digestive – Neurological – Respiratory	6	0.5 (0.2-1.2)
Digestive – Skin – Fever or chills	6	0.5 (0.2-1.2)
Osteoarticular – Osteoarticular – Neurological	6	0.5 (0.2-1.2)
Osteoarticular – Neurological – Skin	6	0.5 (0.2-1.2)
Osteoarticular – Metabolic – Fever or chills	6	0.5 (0.2-1.2)
Neurological – Neurological – Metabolic	6	0.5 (0.2-1.2)
General – Skin – Fever or chills	5	0.5 (0.2-1.1)
Digestive – Neurological – Neurological	5	0.5 (0.2-1.1)
Digestive – Neurological – Metabolic	5	0.5 (0.2-1.1)
Osteoarticular – Osteoarticular – Skin	5	0.5 (0.2-1.1)
Osteoarticular – Skin – Fever or chills	5	0.5 (0.2-1.1)
Osteoarticular – Fever or chills – Fever or chills	5	0.5 (0.2-1.1)
Other	224	20.2

S1afflicted body system related to first expressed reason for consultation. For each proportion, 95% confidence intervals (95% CI) were computed using the Wald method. The sample size for combinations of afflicted body systems is reduced (from n = 1867 to n = 1110) because we only retained patients who expressed at least three reasons for consultation.

#### Distribution by sex and department.

**Fig 3:**
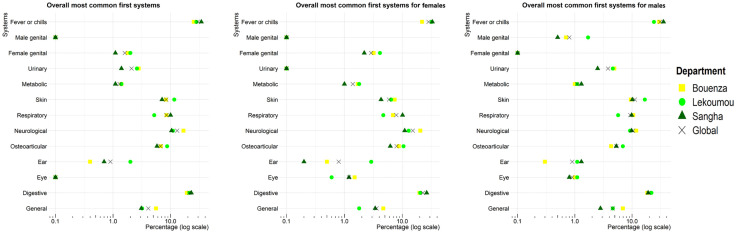
Distribution of the first afflicted body systems stratified by sex and department.

Regarding the afflicted body systems in relation to R_1_ (Fig 3), patients in Bouenza more frequently reported neurological system-related complaints (16.8%) and less frequently reported issues with the Ear-Nose-Throat system (0.4%), compared with patients in Lékoumou (11.0%, and 2.0%, respectively) and Sangha (10.4%, and 0.7% respectively, p < 0.001, and p = 0.030 for neurological and Ear-Nose-Throat based complaints, respectively). In contrast, patients in Lékoumou expressed more frequently skin-related complaints (11.6%), compared with those in Bouenza and Sangha (8.3% and 7.1% respectively, p = 0.043).

#### Distribution by sex and age groups.

**Fig 4:**
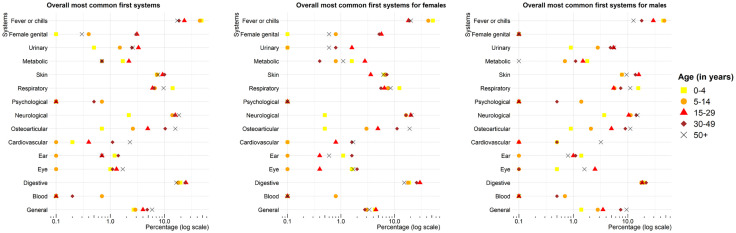
Distribution of the first afflicted body systems stratified by sex and by age group.

The respiratory system (related to R_1_) was more frequently affected in the 0–4-year age group (14.1%), compared with the 5–14-year, 15–29-year, 30–49 year, and 50 years and over age groups (6.6%, 6.0%, 6.4%, and 9.6% respectively, p < 0, 001). The urinary system (related to R_1_) was less frequently affected in 0–4 age group compared with the 5–14-year, 15–29-year, 30–49 year, and 50 years and over age groups (1.5%, 3.3%, 2.5%, and 2.6% respectively, p = 0.048). Furthermore, the osteoarticular system (related to R_1_) was found to be significantly less affected in the 0–4 age group (0.7%) compared with the 5–14-year, 15–29-year, 30–49-year, and 50 years and over age groups (2.6%, 4.9%, 10.3%, and 15.8%, respectively; p < 0.001) (Fig 4).

The distribution of afflicted body systems matched to R1 by department, sex and age showed that the osteoarticular system (see S11 File) was more affected in individuals aged 50 years and over across all three departments (Bouenza: 14.1%, Lekoumou: 16.3%, Sangha: 18.4%), as compared with the age groups 0–4, 5–14, 15–29, and 30–49 years (Bouenza: 0.7%, 1.2%, 5.3% and 10.3%, Lékoumou: 1.8%, 4.2%, 2.9% and 12.3%, Sangha: 0.5%, 2.8%, 5.2%, and 9.3%, p < 0.05). A similar pattern for osteoarticular system was identified across all departments, for all the reasons for consultation that were expressed, irrespective of the order in which the underlying reasons for consultation were expressed (see S12 File). Finally, the distribution of combinations of afflicted body systems related to R_1_-R_2_-R_3_ (see S13 File) revealed that the combination “Respiratory-Respiratory-Fever or chills” was more prevalent in Sangha department (10.2%) as compared with Bouenza and Lékoumou departments (3.5% and 2.7%, respectively), and was more frequent in children aged 0–4-years (20.5%), as compared with participants aged 5–14, 15–29, 30–49, and 50 years and over (5.0%, 3.1%, 1.1%, and 2.1% respectively, p < 0.05).

## Discussion

The objective of this study was to describe the reasons for consultation and afflicted body systems in rural health facilities in three departments of the Republic of the Congo. Fever was the main reason for consultation followed by four reasons for consultation: headache, generalized abdominal pain, cough and diarrhea. Based on the revised ICPC-2 classification, the four most afflicted body systems were, by decreasing order of frequency: “Fever or chills”, digestive system, neurological system, and respiratory system.

Except for a few publications on specialized hospital units, there is a notable lack of comprehensive data on the distribution of reasons for consultation in rural health facilities in sub-Saharan Africa. The limited available data was presented in theses, particularly in West Africa, and on one or two health facilities [29–43]. In Mali, three similar studies on reasons for consultation in general practice, conducted in 2007 [31], 2013–2015 [41] et 2018 [33] reported that fever was the most prevalent reason for consultation (23%, 26.1%, and 25.4%, respectively), followed by headache for the first study (8%), vomiting for the second (10.6%) and abdominal pain for the last study (12.9%). In Cameroon, a prospective study conducted at the maternal and child prevention center between 1988 and 1989 [32] reported that, fever was also the first expressed reason (56.6%), while in mainly primary public healthcare facilities of Senegal (Diourbel region, during the Grand Magal of Touba) [39] in 2016, it was the second reason (17.2%), preceded by headache (28.4%).

In the Republic of the Congo, studies mainly focused on recruited subjects at the Hospital and specialized units of University Center of Brazzaville [44–47]. Two studies conducted in rural areas were identified. The first study was conducted between January 1982 and January 1983 in the Kouilou department and revealed that diarrhea was the primary reason for consultation among children under two years of age (25.2%) and cough (21.0%) among 2–5-year-olds [37]. The second study was conducted in a semi-rural area in close proximity to Brazzaville [48]. In 1981, digestive and respiratory systems accounted for over 50% of consultations in the 0–4 age group, with fever being a common accompanying symptom. Although NTDs are empirically considered to be common diseases in rural areas of the Republic of the Congo, reasons for consultation related to NTDs were uncommon in this study. Despite the paucity of comparable studies, the primary reasons for consultation and afflicted body systems in the overall population and age sub-groups in Sub-Saharan Africa appear to be similar and remained stable over time.

The high proportion of fever can be attributed to the endemic presence of malaria, which accounts for 54% of the causes of consultation in the Republic of the Congo [7], or to the COVID-19 pandemic, for which fever is the most frequent symptom [49–51]. However, the lack of comparable data prevents us from conducting a comparative analysis and accurately estimating the potential impact of COVID-19. The 2019 health report for the countries of the Economic Community of West African State also revealed malaria to be the leading medical cause of consultation, with 38.4% of the general population, 41.7% of children under 5 and 27.0% of adults aged 25 and over affected [30]. Regarding the high proportion of headaches, the description of R_1-3_ combinations (see S10 File) indicated an association with fever in the 30–49 age group, which is likely to be related to an infectious disease. In contrast, the older population had muscle pain or dizziness, and these headaches may be due to several reasons such as cervical arthralgia, tension headaches or arterial hypertension [52,53]. Frequent complaints of abdominal pain were strongly associated with fever and population 0–29 years old, which may suggest benign infections as well as medical-surgical emergencies [54]. While fever and upper respiratory system infection or otorhinolaryngological issues could be the most probable causes, the high frequency of cough may indicate pulmonary tuberculosis and respiratory allergies due to woodworking activities in the logging companies in the Sangha department could explain the higher frequency of cough reported in Sangha [55].

The NTD-related reasons for consultation (rash and pruritus, convulsion, skin swelling, hematuria) were less frequent in reported first reasons for consultation. This may be attributed to the mild nature of these signs and symptoms, which may not require a consultation. Additionally, the decline in morbidity associated with NTDs following the sustained efforts of mass drug administration for numerous NTDs through control programs in these departments may also contribute to this trend. Furthermore, NTDs are mainly chronic diseases, whereas consultations in rural areas are generally for acute pathologies in the Republic of the Congo. This low frequency of NTDs-related symptoms highlights the necessity for active screening for these diseases in the community, as opposed to the collection of data in health facilities, as demonstrated in our methodology.

Our study was not without limitations. Indeed, the participants were not randomly selected, which may have resulted in the findings not fully reflecting the reality of the selected local areas. Furthermore, the refusal of some participants to respond could lead to nonresponse bias, resulting in an underestimation of the reasons for consultation and afflicted body systems. This underestimation could also be observed for signs and symptoms of diseases that are more prevalent during the rainy season, such as malaria [56,57], as the data collection period was restricted to the dry season.. Moreover, the collection period was limited to one month, which may not have been sufficient for patients with chronic conditions. Also, we only reported reasons for consultation, in other words symptoms, while for a given symptom, there are many differential diagnoses. For instance, malaria may not be the only diagnostic of fever, rash can be associated with conditions such as dengue, chikungunya, leishmaniasis, and scabies, whereas pruritus was encountered in cases of onchocerciasis and scabies [58]. In a context where soil-transmitted helminths are endemic, cough may suggest Loëffler’s syndrome [13]. Due to the limitations, we are unable to provide further extrapolations from our data or to discuss all potential diagnostic possibilities. Despite the limitations, the results provide a valuable source of information for public authorities and health care practitioners, as they offer insight into the reasons for consultation and afflicted body systems for which information is often of poorer quality at departmental level and after stratification by age and sex. The choice of the ICPC-2 questionnaire ensured a standardized categorization of health complaints across diverse primary care settings. This enabled internal comparisons between health posts and health centers—which represented most consultations in our study (11.9% and 55.9%, respectively)—and external comparisons with similar research conducted in sub-Saharan Africa [59–61]. Our experience also highlights the practical challenges of data collection in low-resource settings, where electricity shortages and cost constraints often hinder the use of digital tools.

Since people in these rural areas have little or no access to complementary examinations, we recommend that the authorities ensure the production of this information on a regular basis at the national level. Furthermore, it is recommended that health professionals working in rural health facilities guarantee the quality of the data collected in their consultation registries, take ownership of the results of this research, inform the community and provide effective treatment for diseases associated with the afflicted body systems identified in the results.

## Conclusions

This study is the first of its kind in rural areas of the Republic of the Congo. The main reason for consultation was fever, with no difference between departments and “fever or chills” was found to be the most frequently afflicted body system, followed by the digestive system. Rural professionals should be able to well identify digestive signs and pathologies, and referral health facilities should have specialists in this field. To gain a deeper insight into the health status of the rural population in The Republic of the Congo, it is essential to complete these data with the history of diseases, the medical diagnoses made during consultations, and with associated social factors. Furthermore, future similar studies should collect information during rainy seasons as well, given the seasonal nature of several diseases [56,57,62].

## Supporting information

S1 File Project database. (XLSX)

S2 File Strobe checklist. (DOCX)

S3 File Health facilities and geographic coordinates. (XLSX)

S4 File List of abbreviations. (DOCX)

S5 File Population pyramid. (TIF)

S6 File Survey questionnaire. (PDF)

S7 File Selection procedure. (TIF)

S8 File First expressed reasons for consultation from each department. (DOCX)

S9 File All reasons for consultation without order of expression. (DOCX)

S10 File Combination of reasons for consultation stratified by sex, department and age. (DOCX)

S11 File First expressed afflicted body systems from each department. (DOCX)

S12 File All afflicted body systems from each department without order of expression. (DOCX)

S13 File Combination of afflicted body systems related to reasons stratified by sex, department and age. (DOCX)
