# Supplementary material for: Reasons for consultations and afflicted body systems in rural areas of The Republic of the Congo: A cross-sectional study
Source: PLoS One. 2025 Oct 17;20(10):e0333181. doi: 10.1371/journal.pone.0333181 (PMC12533885; doi:10.1371/journal.pone.0333181)
Supplement: S4 File — (DOCX) [file pone.0333181.s004.docx]

**List of abbreviations**

CERSSA: *Comité d’Éthique et de la Recherche en Sciences de la Santé*

CI: Confidence Interval (used for tables 2 to 5)

ICPC: International Classification of Primary Care (used for table 2 and table 4, figures 2 to 5)

NTD: Neglected Tropical Diseases

STH: Soil-transmitted Helminths
