# Supplementary figures and images for: Reasons for consultations and afflicted body systems in rural areas of The Republic of the Congo: A cross-sectional study
Source: PLoS One. 2025 Oct 17;20(10):e0333181. doi: 10.1371/journal.pone.0333181 (PMC12533885; doi:10.1371/journal.pone.0333181)

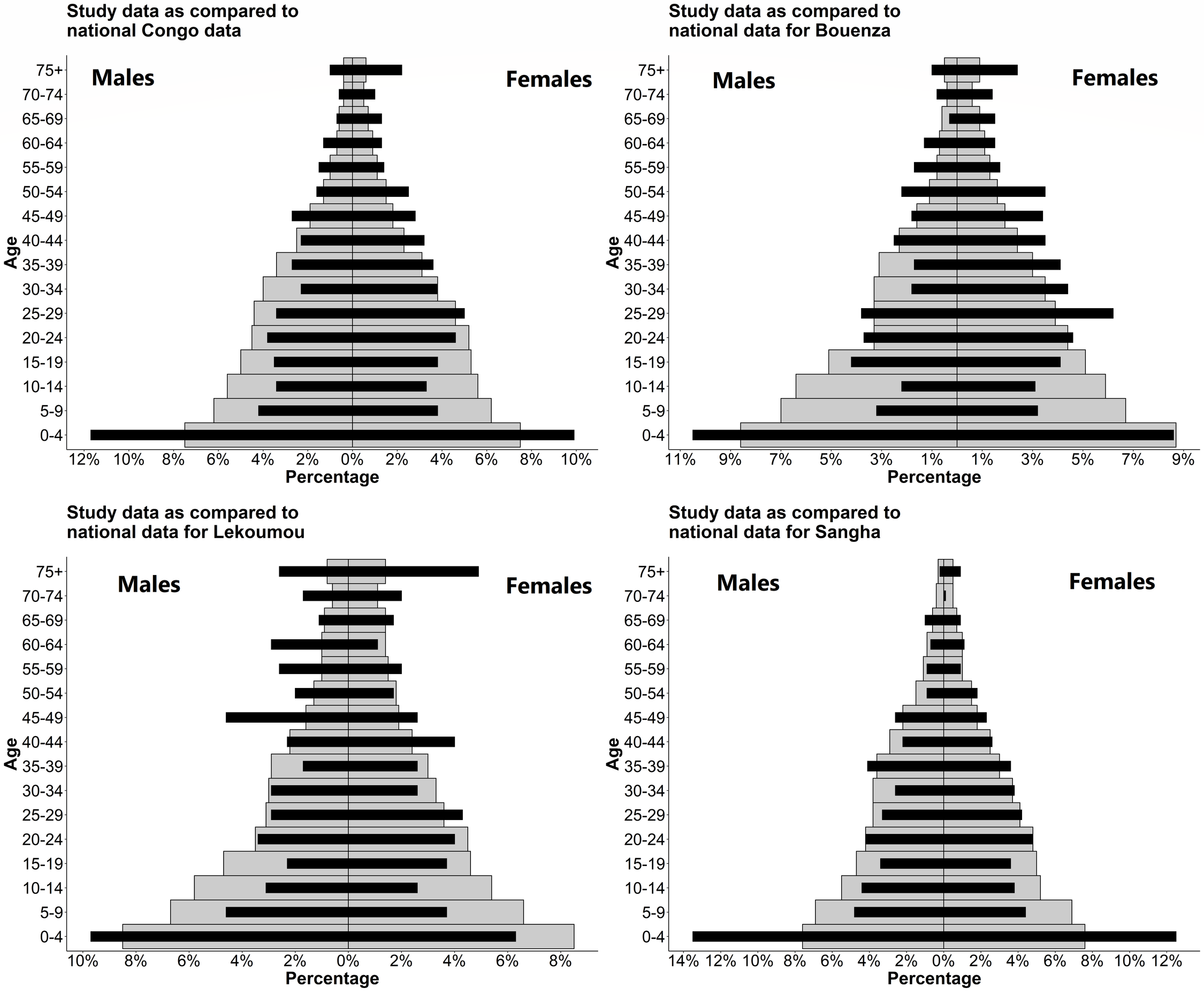

Supplement: S5 File — (TIF) [file pone.0333181.s005.tif]

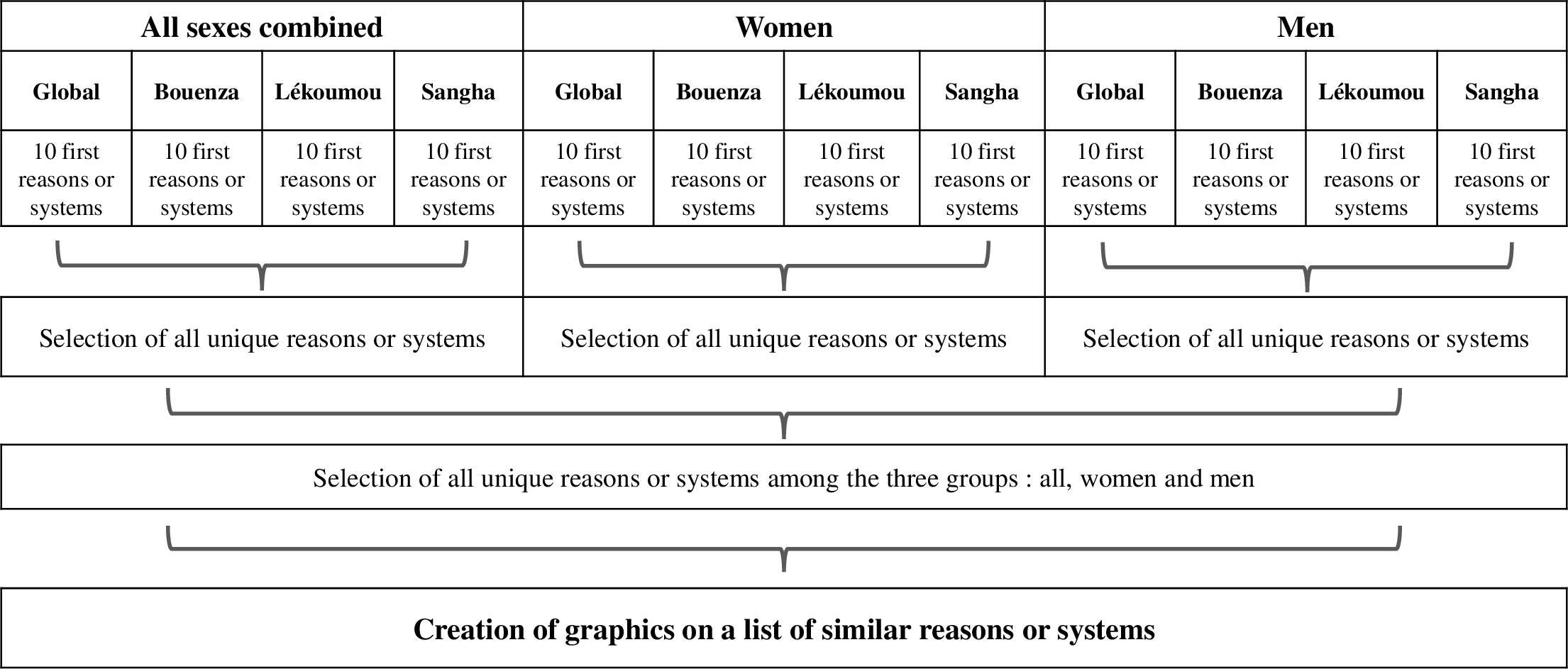

Supplement: S7 File — (TIF) [file pone.0333181.s007.tif]
