# Supplementary material for: Reasons for consultations and afflicted body systems in rural areas of The Republic of the Congo: A cross-sectional study
Source: PLoS One. 2025 Oct 17;20(10):e0333181. doi: 10.1371/journal.pone.0333181 (PMC12533885; doi:10.1371/journal.pone.0333181)
Supplement: S6 File — (PDF) [file pone.0333181.s006.pdf]

Projet de thèse Maladies Tropicales Négligées
Promoteur : OCEAC, KfW ; **Domaine d'étude** : Épidémiologie, Santé publique
Doctorant : NGATSE Joseph Axel (Contacts **:** 06 915 26 05 / 05 389 24 99/ josaxel@yahoo.fr)

Motifs de consultation en zone rurale de la République du Congo :
Questionnaire d'étude

Ce questionnaire permet de recueillir les motifs de consultation dans les formations sanitaires situées en zone rurale, en vue d'identifier les maladies tropicales négligées endémiques en République du Congo. Pour la bonne réalisation de cette étude, nous vous remercions par avance pour le temps de remplissage que vous y consacrerez.

Date de consultation : _____ / _____ / __________

Identifiant du questionnaire (ne pas remplir): /____//____/ - /____//____/ - /____//____//____//____/

Site d'étude

Département : ____

District sanitaire : ____

Centre de santé / Hôpital : ____

Nom et prénoms de l'agent de santé : ____

Patient (e)

Nom et prénoms : ____

Age actuel (en année) : ______________

Sexe : Homme ☐ Femme ☐

Village / quartier de résidence : _____________________________________

Activité professionnelle principale : _____________________________________

Motif(s) de consultation (comme pour le registre de consultation curative)

1. _____________________________ _____________ / __ / __ /
2. _____________________________ _____________ / __ / __ /
3. _____________________________ _____________ / __ / __ /

Début de la maladie et histoire de la maladie (si besoin)

____

____

____

____

____

____
