# Supplementary material for: Reasons for consultations and afflicted body systems in rural areas of The Republic of the Congo: A cross-sectional study
Source: PLoS One. 2025 Oct 17;20(10):e0333181. doi: 10.1371/journal.pone.0333181 (PMC12533885; doi:10.1371/journal.pone.0333181)

**Distribution of all systems (regardless of order) stratified by sex and department**

**
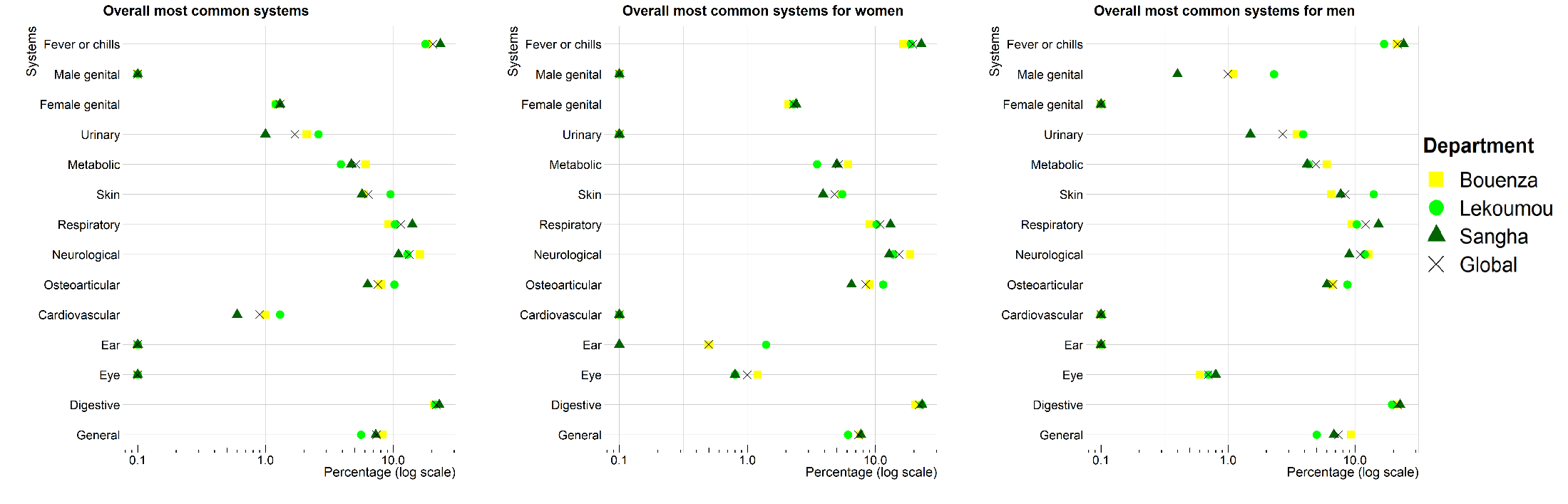
**

**Distribution of all systems (regardless of order) stratified by sex and age group**

**
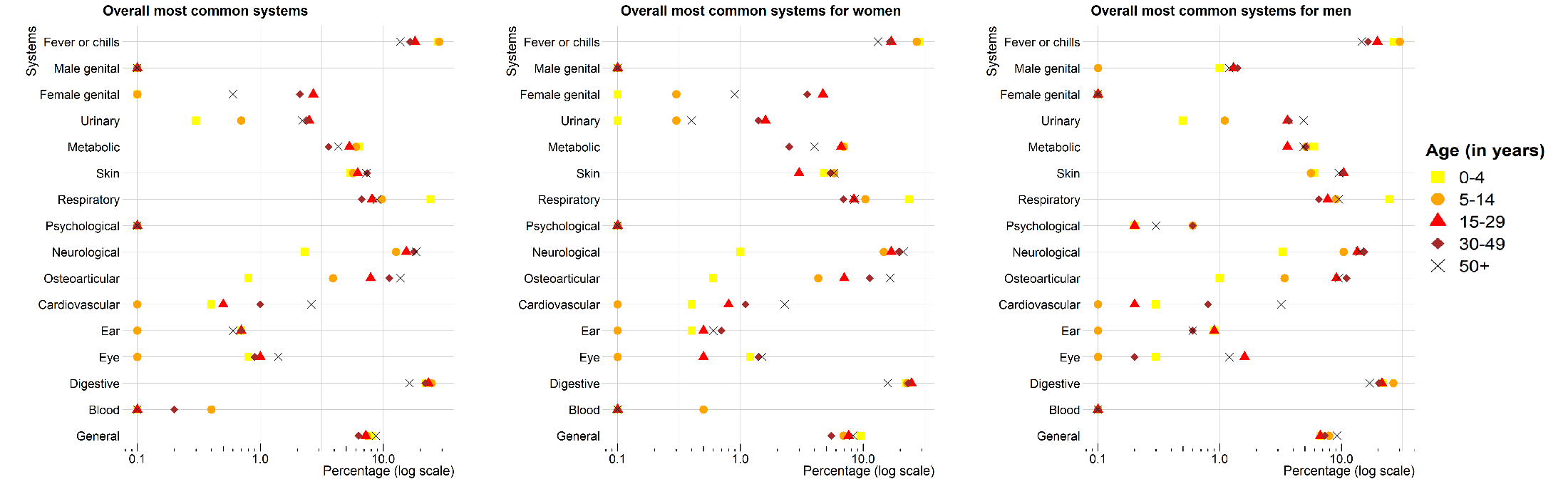
**

**Distribution of all systems (regardless of order) stratified by sex, age group and department**

1. **Bouenza**


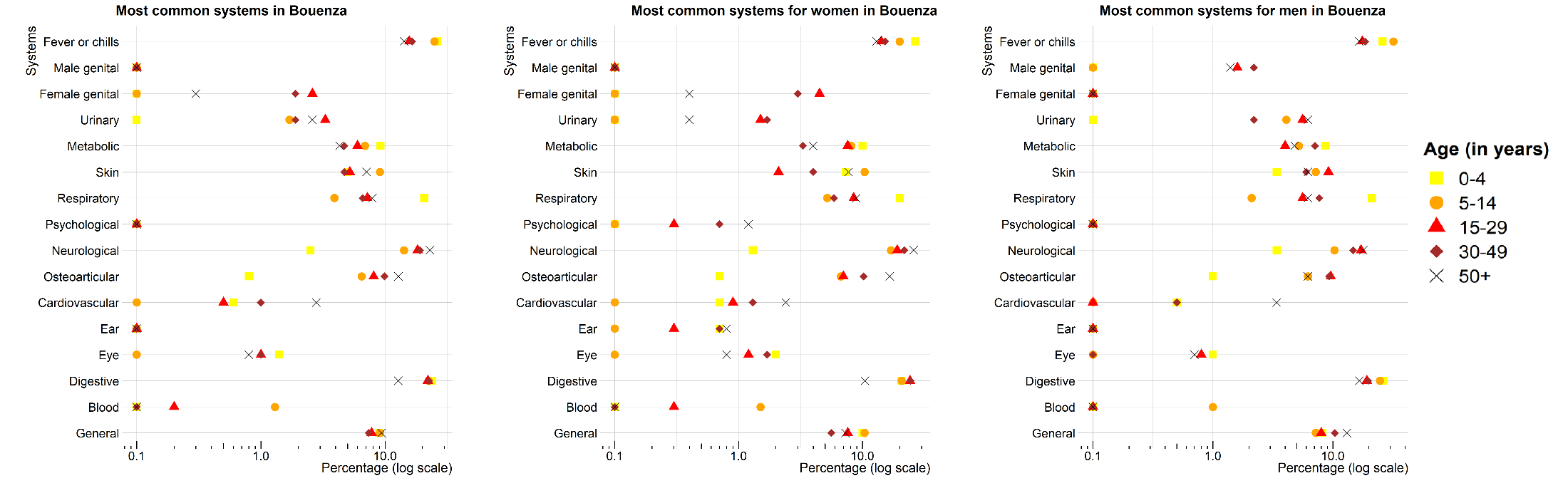


1. **Lékoumou**


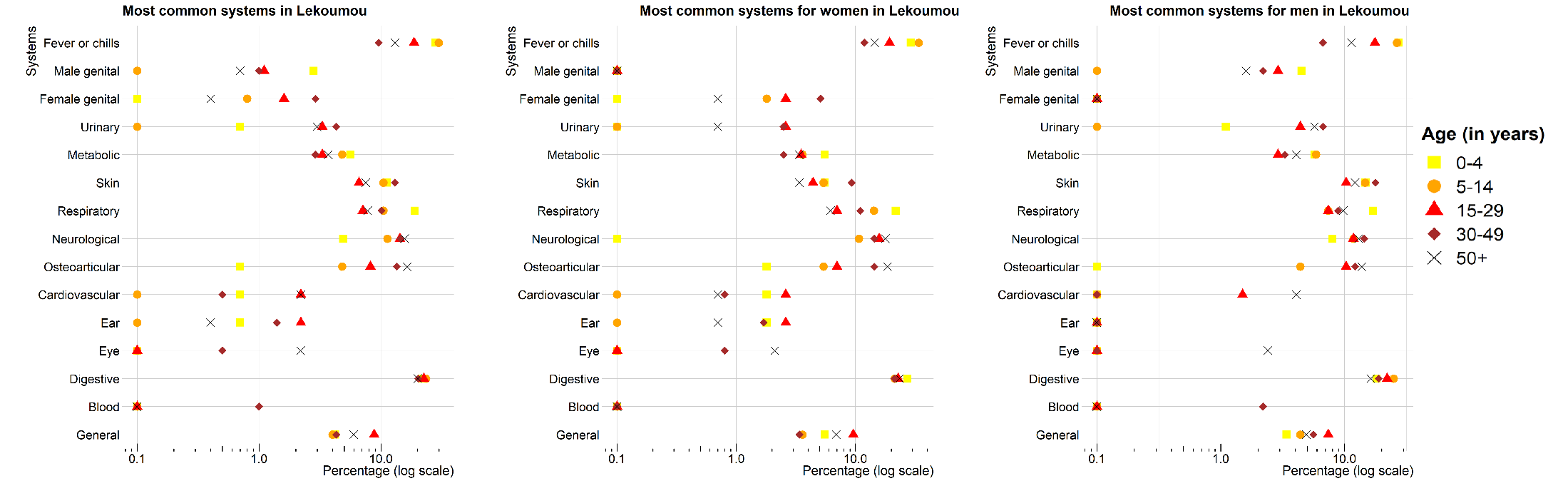


1. **Sangha**


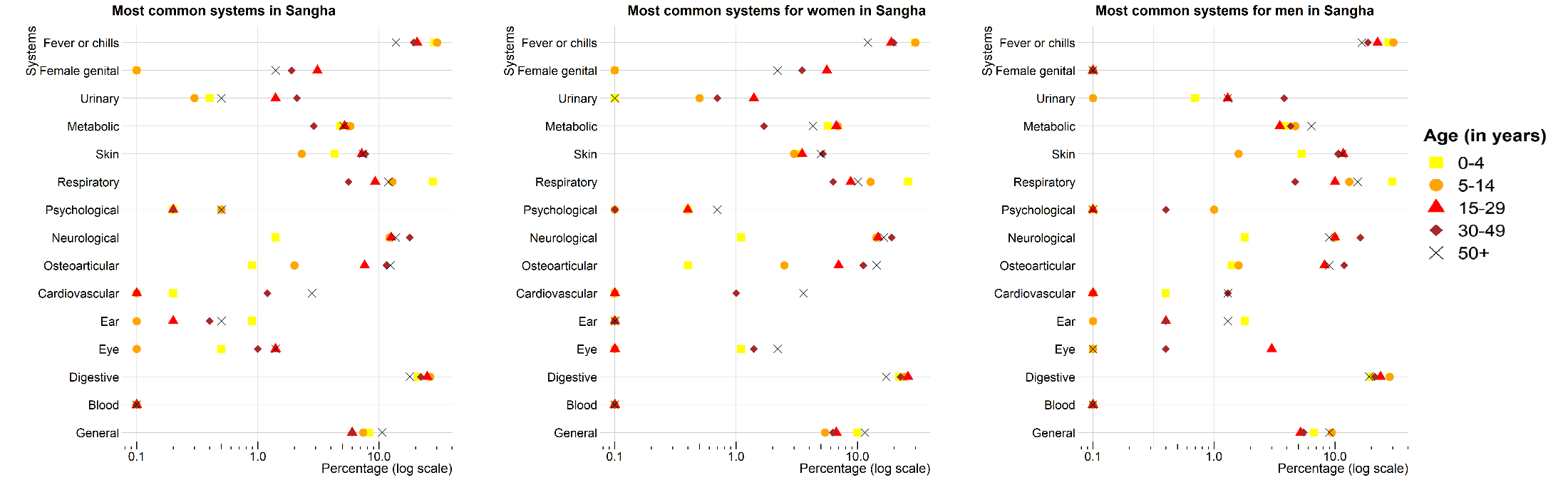

Supplement: S12 File — (DOCX) [file pone.0333181.s012.docx]
