# Supplementary material for: Reasons for consultations and afflicted body systems in rural areas of The Republic of the Congo: A cross-sectional study
Source: PLoS One. 2025 Oct 17;20(10):e0333181. doi: 10.1371/journal.pone.0333181 (PMC12533885; doi:10.1371/journal.pone.0333181)
Supplement: S13 File — (DOCX) [file pone.0333181.s013.docx]

**Distribution of three first system combinations (System 1 - System 2 - System 3) stratified by sex and department**

**
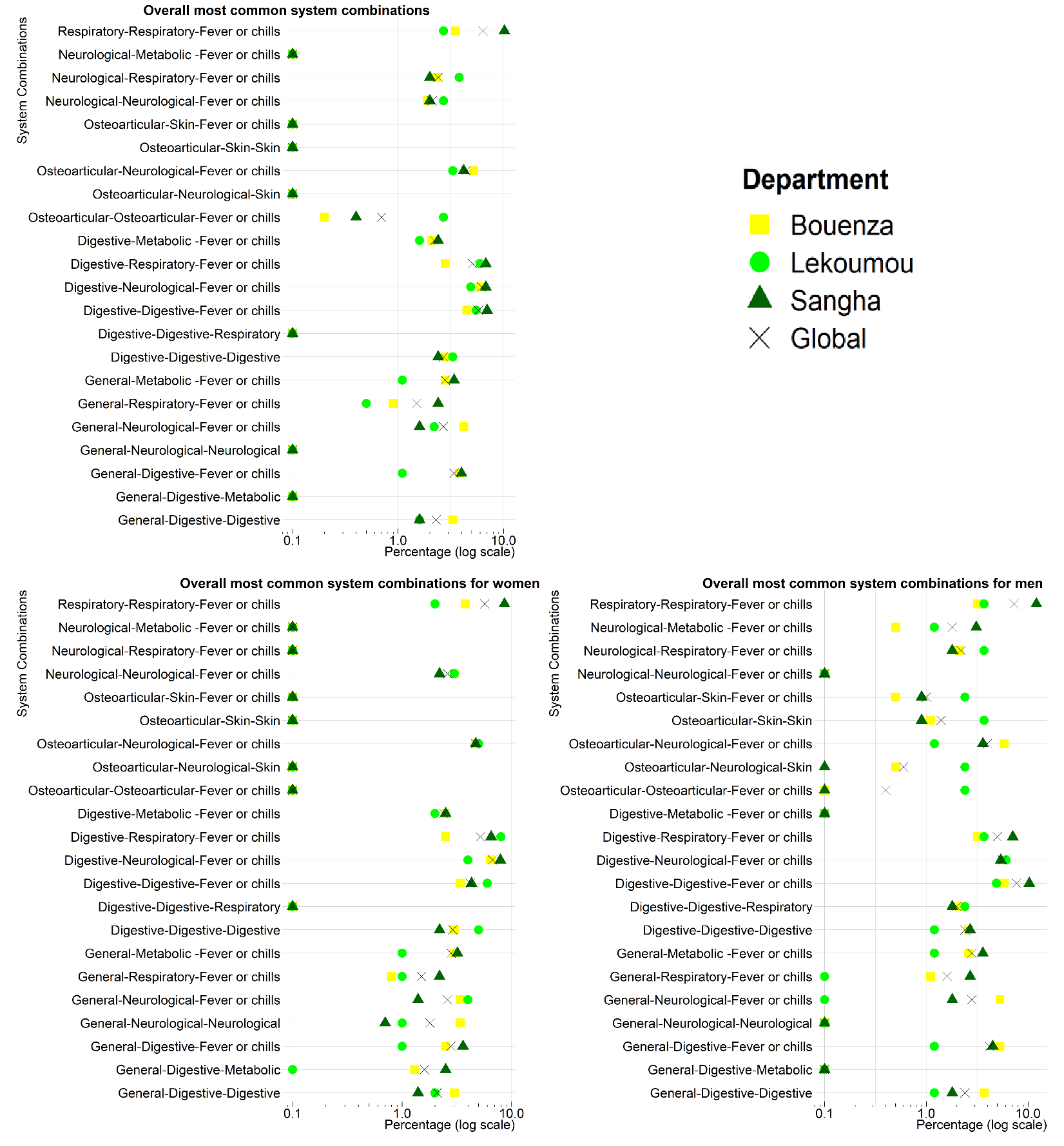
**

**Distribution of three first system combinations (System 1 - System 2 - System 3) stratified by sex and age group**

**
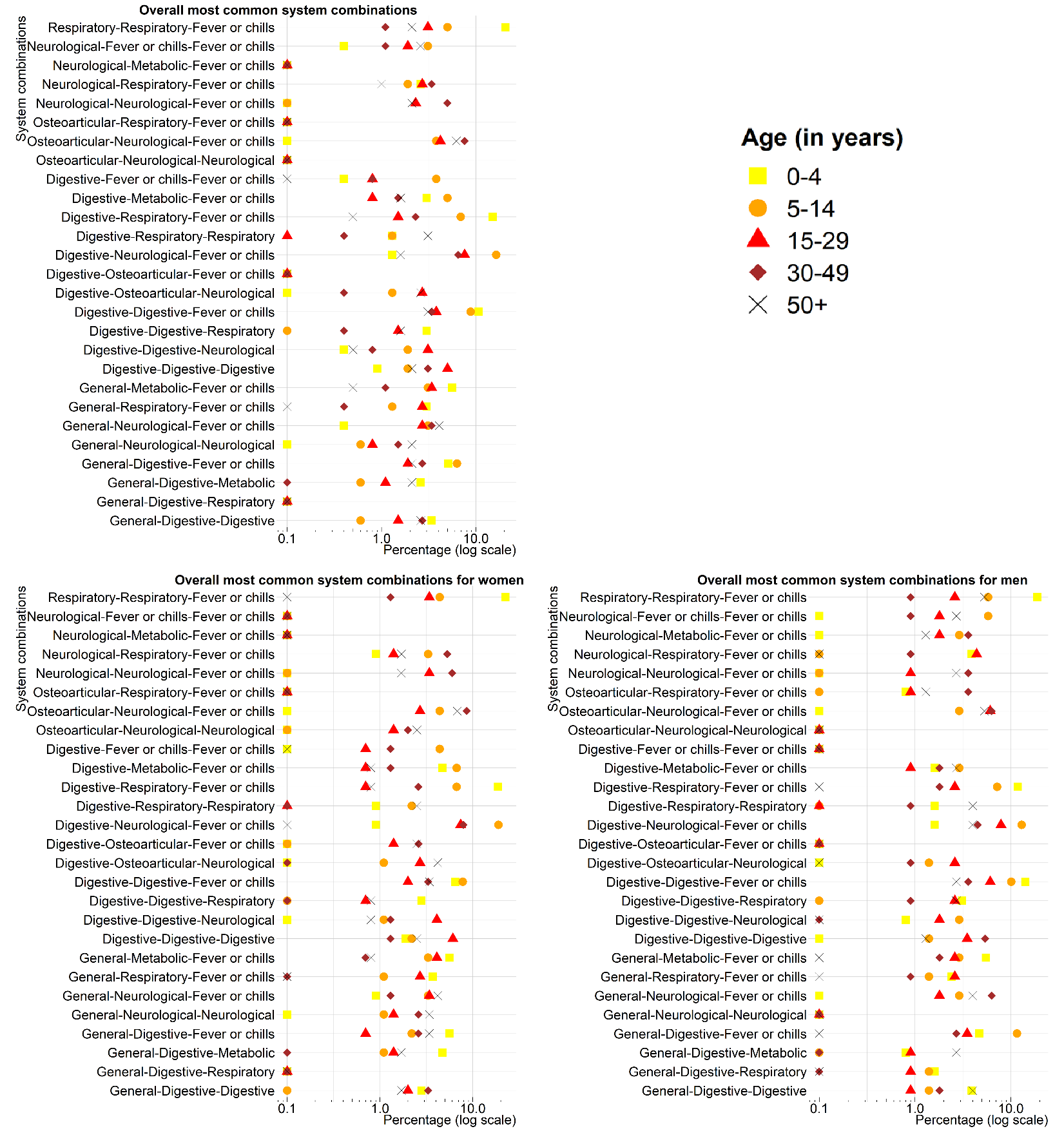
**
